# Combination of mild hypothermia with neuroprotectants has greater neuroprotective effects during oxygen-glucose deprivation and reoxygenation-mediated neuronal injury

**DOI:** 10.1038/srep07091

**Published:** 2014-11-18

**Authors:** Xiao-Ya Gao, Jian-Ou Huang, Ya-Fang Hu, Yong Gu, Shu-Zhen Zhu, Kai-Bin Huang, Jin-Yu Chen, Su-Yue Pan

**Affiliations:** 1 Department of Neurology, Nanfang Hospital, Southern Medical University, Guangzhou, Guangdong, P. R. China; 2 Department of Neurology, Zhujiang Hospital, Southern Medical University, Guangzhou, Guangdong, P. R. China; 3 Department of Neurology, the 421 Hospital, Guangzhou, Guangdong, P. R. China

## Abstract

Co-treatment of neuroprotective reagents may improve the therapeutic efficacy of hypothermia in protecting neurons during ischemic stroke. This study aimed to find promising drugs that enhance the neuroprotective effect of mild hypothermia (MH). 26 candidate drugs were selected based on different targets. Primary cultured cortical neurons were exposed to oxygen-glucose deprivation and reoxygenation (OGD/R) to induce neuronal damage, followed by either single treatment (a drug or MH) or a combination of a drug and MH. Results showed that, compared with single treatment, combination of MH with brain derived neurotrophic factor, glibenclamide, dizocilpine, human urinary kallidinogenase or neuroglobin displayed higher proportion of neuronal cell viability. The latter three drugs also caused less apoptosis rate in combined treatment. Furthermore, co-treatment of those three drugs and MH decreased the level of reactive oxygen species (ROS) and intracellular calcium accumulation, as well as stabilized mitochondrial membrane potential (MMP), indicating the combined neuroprotective effects are probably via inhibiting mitochondrial apoptosis pathway. Taken together, the study suggests that combined treatment with hypothermia and certain neuroprotective reagents provide a better protection against OGD/R-induced neuronal injury.

Ischemic stroke is one of the most common diseases that cause death and disability worldwide, which brings a hard burden to families and society. It starts with sudden cessation of blood flow, oxygen, glucose and energy in the lesion area, followed by series of pathologic cascading events including exitotoxicity, calcium influx, free radicals accumulation, inflammation response, blood-brain barrier breakdown, edema, cell death and so on^1^,^2^.

Over the past several decades, many neuroprotective drugs have been designed to target the different ischemic cascades and prevent the death of salvageable neurons in the ischemic penumbra. Based on the molecular targets, neuroprotective agents are classified and listed examples as:(1)N-methyl-D-aspartate (NMDA) receptor blocker, dizocilpine (MK-801)^3^; (2)γ-aminobutyric acid (GABA) receptor agonist, baclofen^4^; (3)calcium channel blocker, nimodipine^5^; (4)sodium channel blocker, glibenclamide (GBC)^6^, gliclazide^7^; (5)5-hydroxytryptamine receptor agonist, 8-hydroxy-2-(n-dipropylamino)tetralin (8-OH-DPAT)^8^; (6)free radical scavenger, edaravone^9^, vitamine E^10^,VAS2870^11^ and NXY-059^12^; (7)growth factors, brain derived neurotrophic factor (BDNF)^13^; (8)hormones, methylprednisolone (MP)^14^; and (9)other drugs, atorvastatin^15^, progesterone^16^, magnesium^17^, albumin(Alb)^18^, human urinary kininogenase (HUK)^19^, cyclosporine A^20^, minocycline^21^, citicoline^22^, ganglioside^23^, bumetanide^24^, neuroglobulin (Ngb)^25^, and pyruvate^26^. Unfortunately, most drugs proved to be effective in animal studies are failed in clinical trials^27^,^28^. Thus, there is an urgent need for developing novel therapies for stroke.

Therapeutic hypothermia has emerged as a promising neuroprotective therapeutic strategy. It targets multiple ischemic cascades, including energy depletion, excitotoxicity, free radicals, blood-brain barrier breakdown and inflammation. Hypothermia treatment initiated at 31–35°C for 1.5–6 hrs in animal stroke models reduces infarct size and improves neurological behavior^29^,^30^,^31^. Clinically, hypothermia achieves remarkable better outcome in the treatment of cardiopulmonary resuscitation^32^ and neonatal hypoxic-ischemic encephalopathy^33^. Hypothermia with temperate at 33–35.5°C for 2–12 is an effective and feasibility method in acute ischemic stroke in three prospective observation studies or randomized controlled hypothermia trials^34^,^35^,^36^. However, MH treatment after intravenous thrombolysis in patients with acute stroke shows that this treatment does not produce better outcome^36^. Therefore, it is necessary to find the drugs that enhance the efficacy of MH to provide better protective effects for the treatment of ischemic stroke.

Several neuroprotectants have synergistic neuroprotection with MH in animal model of cerebral ischemia, including BDNF^37^, magnesium sulfate^26^ and albumin^18^. Whether other drugs have similar synergistic role is unclear. In our study, 26 drugs were selected based on the different targets,most of which have undergone phase I or II clinic trials. Oxygen-glucose deprivation and reoxygenation (OGD/R) was used to induce the neuronal injury model in primary cultured cortical neurons to mimic the brain ischemia in vitro. With this model, we compared the neuroprotective effects of the 26 candidate medicines with or without MH. Among them, compared with single treatment, HUK, MK-801 or Ngb were shown to have better protective effects in combination with hypothermia against OGD/R-induced neuronal damage.

## Results

### Screening neuroprotective reagents having better protective effects in combination with MH

**Figure 1:**
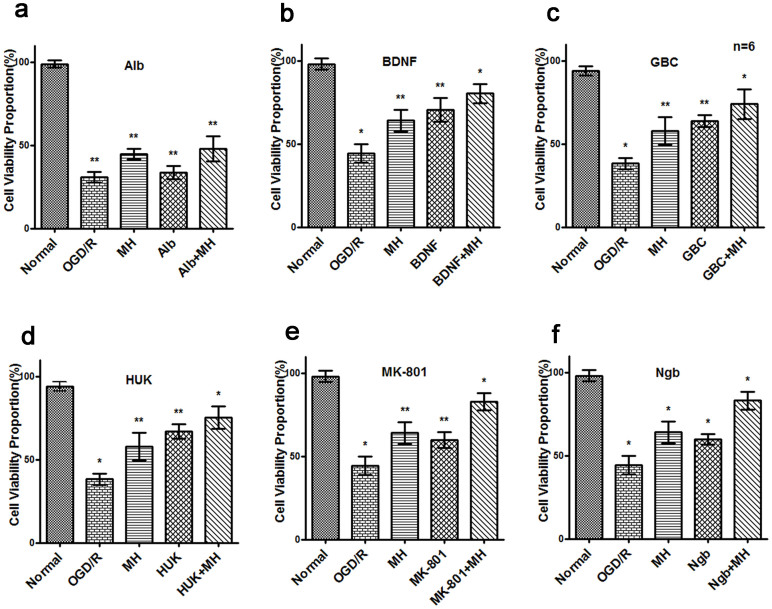
Combination of neuroprotectants and mild hypothermia had better protection effect on primary cultured neurons after OGD/R. The cultured cortical neurons (DIV 6) were subjected to OGD for 3 hrs. While glucose recovery and reoxygenation initiated, cells were immediately treated with a drug (24 hrs), mild hypothermia (MH, 4.5 hrs at 34°C, then 19.5 hrs at 37°C), or combination of both of them. Cells without OGD/R were as normal control. CVP was measured with the CCK-8 Assay Kit (Dojindo Laboratories, Tokyo, Japan) before OGD (CV_pre_) and 24 hrs after reoxygenation (CV_post_). CVP = CV_post_/CV_pre_ × 100%. The bars represent means ± s.d. (n = 6).*, P<0.05 compared with the other groups, and **, P<0.05, compared with one or two groups. Among 26 drugs, BDNF, MK-801, GBC, HUK and Ngb, combined with MH presented more efficient protection against OGD/R-induced damage (indicated as b–f). But others had no additional protection with hypothermia, represented by albumin. (a). The CVP of combination of 5% albumin with MH was higher than albumin (t = −4.027, P = 0.002) but not MH alone (t = −0.926, P = 0.376). (b). The CVP of combination of 25 ug/ml BDNF with MH was higher than BDNF (t = −2.564, *P* = 0.028) or MH alone (t = −4.479, *P* = 0.001). (c). The CVP of combination of 1 μM GBC with MH was higher than GBC (t = −2.597, *P* = 0.027) or MH alone (t = −3.210, *P* = 0.009). (d). The CVP of combination of 0.0015 PNA/ml HUK with MH was higher than HUK (t = −2.484, *P* = 0.032) or MH alone (t = −3.927, *P* = 0.003). (e). The CVP of combination of 10 μM MK801 with MH was higher than MK801 (t = −8.064, *P*<0.001) or MH alone (t = −5.518, *P*<0.001). (f). The CVP of combination of 50 nM Ngb with MH was higher than Ngb (t = −9.400, *P*<0.001) or MH alone (t = −5.509, *P*<0.001). Abbrevation: BDNF: brain-derived neurotrophic factor; GBC: glibenclamide; MK-801: dizocilpine; HUK: human urinary kininogenase; Ngb: neuroglobin; OGD/R: oxygen glucose deprivation and reperfusion; DIV: days in vitro; s.d.: standard deviation; CV: cell viability; CVP: cell viability proportion.

To search drugs that have synergistic neuroprotective effects with MH, 26 drugs were firstly selected in combination with MH to treat primary cultured cortical neurons challenged with 3 hrs OGD and 24 hrs reoxygenation. Working concentrations of each drug were indicated in Table 1. The purity of mature neurons was more than 97% as assayed by staining of antibody against Neuronal Class III β-tubulin, a specific marker of neurons (data not shown). As shown in figure 1, OGD/R induced a dramatic reduction of cell viability proportion (CVP), while treatment of MH or six drugs, including Alb, BDNF, GBC, HUK MK801 and Ngb, significantly recovered CVP (*P*<0.05). Furthermore, combination of MH with each of above drug, except Alb, displayed higher CVP compared with each single treatment (*P*<0.05). We conducted two-way ANOVA and found that MH and four drugs (BDNF, GBC, MK801 or Ngb) have interactions (*P*<0.05). These results suggest that combination of MH with BDNF, GBC, HUK MK801 and Ngb has greater protective effects during OGD/R-mediated neuronal CVP reduction.

The remaining 21 reagents presented no additional protective effects combined with MH, compared to single treatment (data not shown).

### Combination of MH with HUK, MK-801 or Ngb prevented neuronal apoptosis in OGD/R model

**Figure 2:**
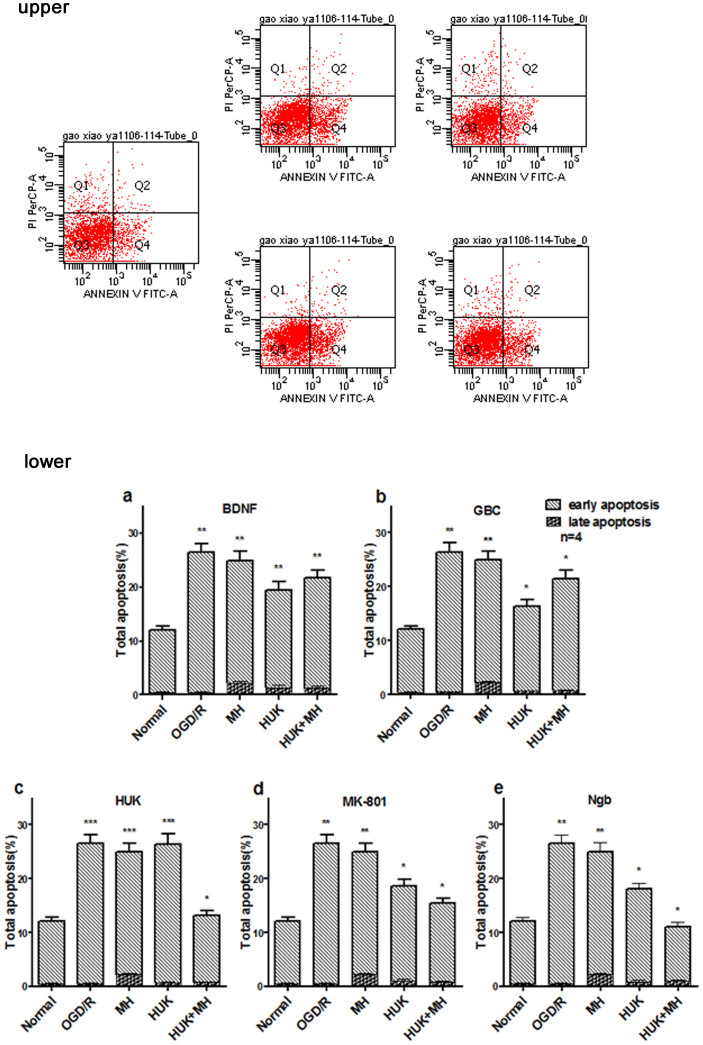
Combination of MH with HUK, MK-801 or Ngb prevented neuronal apoptosis against OGD/R injury. Upper panel: Population analysis or cells with Annexin V/PI Staining. The percentages of apoptosis cells (Q2+Q4) in primary cortical neurons after OGD/R treated by MH combined with HUK, were less than MH or HUK alone. The primary cortical neurons were cultured in 6-well plates. In DIV6, cells were subjected to OGD for 3 hrs, then treated with HUK, HT, or both of them, and reoxygenation were simultaneously made. 48 hrs later, apoptosis was measured with the Annexin V/PI Staining Assay Kit (Sigma, St. Louis, MO) and detected by FACS. As showed on upper panel, necrosis cells stained by propidium iodide (PI) appeared in the first quadrant, late apoptosis cells stained by annexin V-FITC and PI were in the second quadrant, alive cells in the third quadrant, and the early apoptosis cells stained by annexin V-FITC presented in the fourth quadrant. Lower panel: Combinations of each of three Neuroprotectants and MH Prevented OGD/R Neurons from Apoptosis. Processing of primary cortical neurons and analysis of apoptosis by flowcytomery were performed as above. This figure illustrated bar graphs of apoptosis analysis results. The bars represented population percentage by means ± s.d. (n = 4). *, P<0.05 compared with the other groups, and **, P<0.05, compared with one or two groups. Three reagents, HUK, MK-801 and Ngb, combined with MH prevented much more greatly against the apoptosis of primary cerebral neurons after OGD/R, compared with single agent (P< 0.05). (a) The co-treatment of BDNF and MH reduced the total apoptosis percentage, was less than MH (t = 2.561, *P* = 0.043) but not BDNF (t = −1.867, *P* = 0.111). (b) The co-treatment of GBC and MH reduced the total apoptosis percentage, was less than MH (t = 2.804, *P* = 0.031) but more than GBC (t = −4.594, *P* = 0.004). (c) The co-treatment of HUK and MH reduced the total apoptosis percentage, was less than HUK (t = 11.504, *P*<0.001) or MH (t = 11.360, *P*<0.001). (d) The co-treatment of MK-801 and MH reduced the total apoptosis percentage, was less than MK-801 (t = 4.031, *P* = 0.007) or MH (t = 9.196, *P*<0.001). (e) The co-treatment of Ngb and MH reduced the total apoptosis percentage, was less than Ngb (t = 9.504, *P*<0.001) or MH (t = 13.595, *P*<0.001). Abbrevation: Q: quadrant; FACS: Fluorescence Activated Cell Sorter.

We next confirmed the combined protective roles of drugs and MH through calculating the apoptosis rate with Annexin V/PI staining by flow cytometery. As shown in the upper panel of figure 2, the combined treatments of HUK, MK-801 or Ngb with MH displayed more reduction of both early and late apoptosis percentage after OGD/R than each single treatment (*P*<0.05).Combination of MH and BDNF or GBC decreased the apoptosis rate, but did not reduced more than either single treatment (P>0.05). Two-way ANOVA indicated there was interaction between each of these neuroprotectants and MH on apoptosis of neurons after OGD/R. Therefore, combination of HUK, MK-801 or Ngb with MH has greater anti-apoptosis effects.

### Combined of MH with HUK,MK-801 or Ngb significantly reduced the level of intracellular ROS and calcium, and stabilized MMP after OGD/R

We then investigated the relevant mechanisms involved in the greater anti-apoptosis effects of the combo of drugs and MH. Neurons challenged with OGD/R have higher level of intracellular reactive oxygen species (ROS) and calcium influx, as well as reduced mitochondrial potential (MMP, ΔΨm), all of which are landmarks of neuronal apoptosis and play important roles in cerebral ischemia and reperfusion^2^.

**Figure 3:**
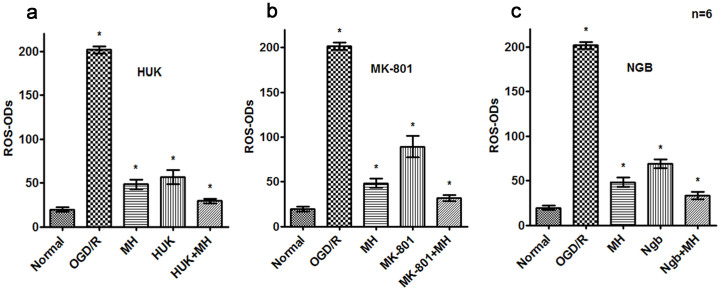
Co-treatments reduced reactive oxygen species (ROS) generation after OGD/R. Processing of primary cortical neurons were performed as above. The ROS were detected with DCFH-DA (Sigma, St. Louis, MO) staining by Spectra Max M5 Multi-Detection Microplate Readers (Molecular Devices, Sunnyvale, CA). The bars represent means ± s.d. (n = 4).*, P<0.05 compared with the other groups, and **, P<0.05, compared with one or two groups. MH combined with HUK,MK-801 or Ngb significantly reduced intracellular ROS generation after OGD/R. (a) The combination of HUK and MH reduced the ROS generation of primary cortical neurons after OGD/R, and was superior to HUK alone (t = 8.055, *P*<0.001) or MH alone (t = 7.849, *P*<0.001). (b) The combination of MK-801 and MH reduced the ROS generation of primary cortical neurons after OGD/R, and was superior to agent (t = 11.581,*P*<0.001) or MH alone (t = 6.542, *P*<0.001). (c) The combination of Ngb and MH reduced the ROS generation of primary cortical neurons after OGD/R, and was superior to agent (t = 13.569, *P*<0.001) or MH alone (t = 5.356, *P*<0.001).

Intracellular ROS was stained with specific probe DCF-DA and quantified according to optical densities (ODs). Results showed OGD/R significantly increased ROS generation, whose level was more than 10 times than that of normal cells (*P*<0.01, Figure 3). Moreover, the combined treatments of HUK, MK-801 or Ngb with MH decreased intracellular ROS after OGD/R, with the reduction extent more than any single treatment (*P*<0.05, Figure 3). Statistical analysis indicated that there were interaction between MK-801 or Ngb and MH on ROS level in primary neurons after OGD/R according to the result of two-way ANOVA (*P*<0.05).

**Figure 4:**
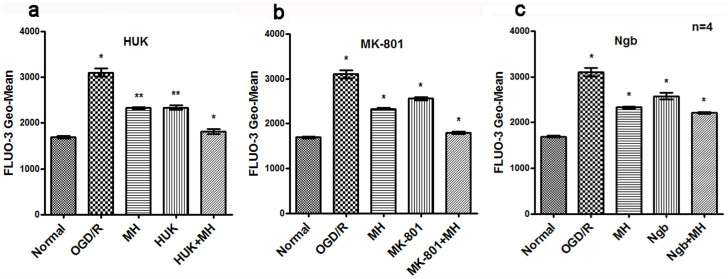
Co-treatments reduced intracellular free calcium concentration ([Ca^2+^]_i_) after OGD/R. Processing of primary cortical neurons were performed as above. The intracellular calcium concentration was determined with Fluo-3 (Biotium, Hayward, CA) staining and detected by FACS. The bars represent means ± s.d. (n = 4).*, P<0.05 compared with the other groups, and **, P<0.05, compared with one or two groups. MH combined with HUK,MK-801 or Ngb significantly decreased [Ca^2+^]_i_ after OGD/R. (a) The combination of HUK and MH reduced the intracellular calcium concentration in primary cortical neurons after OGD/R, to 1,817.500 ± 51.157, was superior to HUK alone (t = 14.901, *P*<0.001) or MH alone (t = 18.294, *P*<0.001). (b) The combination of MK-801 and MH reduced the intracellular calcium concentration in primary cortical neurons after OGD/R, was superior to MK-801 alone (t = 35.284, *P*<0.001) or MH alone (t = 30.352,*P*<0.001). (c) The combination of Ngb and MH reduced the intracellular calcium concentration in primary cortical neurons after OGD/R, was superior to Ngb alone (t = 9.374, *P* = 0.004) or MH alone (t = 7.101, *P* = 0.002). Abbrevation: [Ca^2+^]_i_ :intracellular free calcium concentration.

Intracellular calcium level ([Ca^2+^]_i_) was measured by calcium sensor FLUO-3 AM and analyzed by Fluorescence Activated Cell Sorter (FACS). As shown in Figure 4, OGD/R significantly increased the [Ca^2+^]_I_ relative mean fluorescence intensity, compared with normal group (*P*<0.01). The combined treatments of HUK, MK-801 or Ngb with MH had more reduction of [Ca^2+^]_i_ after OGD/R than single treatment (*P*<0.05, Figure 4). There were interactions only between HUK or Ngb and MH on [Ca^2+^]_i_ according to two-way ANOVA (*P*<0.05).

**Figure 5:**
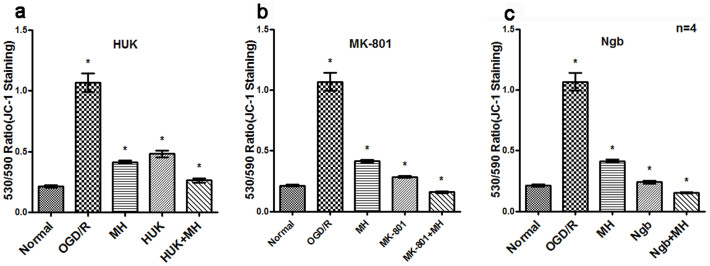
Co-treatments protected Mitochondrial Membrane Potential after OGD/R by reducing 530/590 ratio. Processing of primary cortical neurons were performed as above. The ratio of the neurons with depolarized MMP to that with normal MMP was determined with JC-1 (Beyotime, Shanghai, China) staining and by FACS. Red staining (λ_ex_: 520 nm; λ_em_: 590 nm) represented JC-1 aggregation with normal MMP, green staining (λ_ex_: 490 nm; λ_em_: 530 nm) represented JC-1 monomer with depolarized MMP. The bars represent means ± s.d. (n = 4). *, P<0.05 compared with the other groups, and **, P<0.05, compared with one or two groups. MH combined with HUK,MK-801 or Ngb significantly decreased 530/590 ratio in OGD/R neurons. (a) The combination of HUK with MH decreased green/red (530/590) ratio, and was less than that treated with HUK (t = 37.534, *P*<0.001) or MH alone (t = 12.352, *P*<0.001). (b) The combination of MK-801 with MH decreased green/red (530/590) ratio, and was less than that treated with MK-801 (t = 33.216, *P*<0.001) or MH alone (t = 21.345, *P*<0.001). (c) The combination of Ngb with MH decreased green/red (530/590) ratio, and was less than that treated with Ngb (t = 14.795, *P*<0.001) or MH alone (t = 13.473, *P*<0.001). Abbrevation: ex: excitation, em: emission.

Finally, we compared MMP in different treatment groups measured by JC-1 probe. Results indicated that OGD/R significantly decreased MMP of neurons, showing increased green/red staining (λ_ex_/λ_em_) ratio in cells in the Figure 5 (*P*<0.01). Combined treatment of HUK, MK-801 or Ngb with MH increased MMP after OGD/R, displaying lower green/red signaling than each single treatment (*P*<0.05, Figure 5). There were interactions between MK-801 or Ngb and MHon MMP in primary neurons after OGD/R, according to two-way ANOVA analysis (*P*<0.05). These results, when taken together, suggest that the additional neuroprotective effects of the combined treatments of MH with HUK, Ngb or MK-801 are related to the reduction of calcium influx, intracellular level of ROS and stabilization of MMP.

## Discussion

In this study, through screening 26 protective drugs, we are the first to identify HUK, Ngb, or MK-801, in combination with MH, greatly protected neurons against OGD/R-induced apoptotic cell death. Co-treatment of MH with HUK, Ngb or MK-801 induced greater inhibitory effects on ROS formation, intracellular calcium accumulation and mitochondrial depolarization, compared with either single treatment. The combination of MH with HUK, MK-801 or Ngb prevented neurons against OGD/R-induced apoptosis, probably through mitochondrial apoptosis pathway.

HUK, MK-801 and Ngb, presenting additional protection with hypothermia belong to kallikrein, non-competitive NMDAR blocker and nerve peptide, respectively. HUK, a serine protease isolated from human urine, is approved for the treatment of ischemia stroke in China. It can catalyze kininogen into bradykinin which binds to bradykinin B2 receptor^19^. HUK can produce anticoagulation effect^38^, promote vascular dilation in infarcted zone, boost endothelial cell proliferation^39^, inhibit neuronal apoptosis^19^ and thereby protecting the ischemic brain. Our study provides additional neuroprotective role of HUK that enhances the effect of hypothermia.

MK-801 was reported to be effective in reversible focal ischemia in rats^40^. Unfortunately, it caused severe side effects in clinical study^41^. It might be better if lower dosage and combined therapy employed. A previous study showed the MK-801/hypothermia combination treatment group displays better neuronal protection in hypoxic-ischemic brain injury in neonatal rats^42^. Another study reported they have no additional protective effect in traumatic brain injury in neonatal rats^43^. We found 10 μM MK-801, in combination with MH, had better neuroprotective effect than any single treatment. This discovery might extend the animal study and clinic usage of MK-801 in the future.

Ngb was shown to regulate oxygen molecule delivery^44^ and protected neurons in the model of ischemic stroke^45^. The combined role of Ngb and hypothermia has not been tested.

In summary, our study proved HUK, MK801 and Ngb were good candidates for the combination therapy with hypothermia during OGD/R-mediated neuronal injury.

Animal studies have demonstrated several drugs combined with hypothermia have synergistic neuroprotective effects during cerebral ischemia and reperfusion injury, for example, MgSO_4_^46^, BDNF^47^ and Alb^48^. Nevertheless, we did not found the better protective effects of these three drugs in cultured neurons compared with single treatment. The inconsistency of the in vitro and in vivo data might due to the cultured neurons lack the specific microenvironment composed of vascular system, stem cells, glia cells and other supporting cells. With the complicated environment in vivo, drugs have additional indirect pathway to protect the brain. For instance, MgSO_4_ can dilates blood vessels^17^, while BDNF can promote neurogenesis^47^. We evaluate combined treatments only in cultured neurons which lose interaction with glia cells, endothelial cells and pericytes in neurovascular unit during ischemic stroke. Thus, limitation of the in vitro model used in our study may induce false-negative data.

Another typical example is the combination of GBC with hypothermia. GBC blocks Sur1/trpm4, a Na^+^ channel located mainly expresses in endothelial cells, and thereby reducing oncotic endothelial cell death^49^,^50^. We found the combined effect of GBC with MH on CVP but not on apoptosis rate. Nevertheless, our unpublished data showed that GBC had synergistic protection with hypothermia in a rat model of cerebral ischemia. The neuronal apoptosis rate but not endothelial oncotic cell death was determined in this study might explain this inconsistency. It might also be due to the giant discrepancy of the environment in cell and animal system. Therefore, it is necessary to further evaluate the combined effect of five candidates or even more drugs with hypothermia in animal models.

In conclusion, we identify three drugs, HUK, Ngb and MK-801, provides better protection in combination with hypothermia on cortical neurons against OGD/R-induced damage. Our study provides a new cue for understanding the combination role of drugs and hypothermia, and may offer new approach for clinical practice.

## Methods

### Ethics statement

All experiments in our study were approved and carried out in accordance with the Institutional Animal Care and Use Committee of the Laboratory Animals Center, Nanfang Hospital, Southern Medical University.

### Primary culture of cortical neurons and immunocytochemistry

Primary cortical neurons were cultured as previously described^51^. Briefly, brains from 2 to 3-days-old postnatal Sprague-Dawley (SD) rats were isolated, minced, dissolved, filtered, and then suspended in the DMEM medium (Life Tech, Grand Island, NY) containing 10% fetal bovine serum (Life Tech). The suspended cells were then seeded on plates (3–5 × 10^5^/ml) coated poly-D-lysine (Sigma, St. Louis, MO) and cultured at 37°C in humidified air with 5% CO_2_. After 2–4 hrs incubation, the medium was replaced by neuronal cultured medium containing Neurobasal A(Life Tech) supplemented with B27 (Life Tech) and L-glutamine (Life Tech). The cells were cultured for additional 6 days (DIV 6). The purity of mature neurons was determined by immunostaining with the antibody against Neuronal Class III β-tubulin (1:1000, Beyotime, Shanghai, China), a specific marker of mature neurons.

### OGD/R

The DIV 6 cortical neurons were exposed to OGD/R. Briefly, cultured medium was replaced by glucose-free Neurobasal A medium (Life Tech) and the cultured neurons were put in a hypoxic chamber at 37°C (Thermo Fisher, Waltham, MA) with a mix gas containing 5% CO_2_, 1% O_2_ and 94% N_2_ to reach final 2% O_2_, which was monitored with O_2_ analyzer (GODEE, China). After 3 hrs OGD, neurons were returned to normal cultured conditions for 24 or 48 hrs reoxygenation. The time of management can be found as Supplementary Chart 1 online.

### Treatments of MH and drugs

MH treatment was achieved by placing cultured cells in the cell culture incubator at 34°C environment with 5% CO_2_. After 3 hrs OGD, cells were placed at 34°C for 4.5 hrs and then returned to regular cell incubator for additional 19.5 hrs or 43.5 hrs. After OGD completed, neurons were immediately treated with drugs for 24 hrs or 48 hrs. The drugs used in our study were listed in Table 1. Three or more concentrations were selected around the concentration reported by literature.

### Measurement of cell viability with Cell Counting Kit-8 (CCK-8)

Cell viability was measured with CCK-8 (Dojindo Laboratories, Tokyo, Japan) according to the instructions of the manufacturers. In brief, DIV 6 cortical neurons were seeded in 96-well plates with 150,000 cells/well in the proper medium described above. Six wells were prepared for each treatment or control. 10 µl of the CCK-8 mixture containing 2-(2-methoxy-4-nitrophenyl)-3-(4-nitrophenyl)-5-(2,4-disulfophenyl)-2H-tetrazolium,monosodium salt (WST-8) solution was added to each well containing 100 µl medium. Cells were incubated at 37°C for 3.5 hrs. The ODs were measured at 450 nm with Spectra Max M5 Multi-Detection Microplate Readers (Molecular Devices, Sunnyvale, CA). 24 hrs after OGD or treatments, the ODs were measured again. Cell viability proportion (CVP) was determined by dividing ODs after OGD or treatments by ODs before OGD timed by 100%.

### Measurement of apoptosis with Annexin V/PI staining

Flow Cytometery was used to count apoptotic cells with Annexin V-FITC Apoptosis Detection Kit (Sigma, St. Louis, MO). In brief, 48 hrs after OGD or treatment, neurons were harvested, washed and resuspended in binding buffer at a cell concentration of 1 × 10^6^/ml. For each 500 μl cell suspension, 5 μl Annexin V-FITC and 10 μl of propidium iodide were added, followed by detection with FACS (canto II, Becton, Dickinson and Company, Franklin Lakes, NJ).

### Detection of ROS

24 hrs after OGD, cells were incubated with 2′,7′-dichlorofluorescin diacetate (DCF-DA, 20 µM/ml, 100 µl/well, Sigma, St. Louis, MO) in phenol red-free DMEM for 20 min at 37°C in the dark. DCF-DA, a non-fluorescent compound, can be oxidized by ROS into 2′, 7′-dichlorofluorescin (DCF), a highly fluorescent compound, whose signaling was observed by the Multi-Detection Microplate Readers with excitation wavelength at 488 nm and emission wavelength at 525 nm.

### Detection of [Ca]_i_ and MMP

24 hrs after OGD, neurons were assessed for intracellular calcium concentration and mitochondrial membrane potential (MMP). For calcium concentration detection, neurons were incubated with 1 µM calcium sensor FLUO-3 AM (Biotium, Hayward, CA) dissolved in DMSO and 20% Pluronic F-127 (Biotium, Hayward, CA) for 30 min at 37°C and analyzed with FACS (excitation: 488 nm; emission: 526 nm). To determine MMP, neurons were incubated with JC-1 (Beyotime, Shanghai, China). JC-1 forms a monomer at low MMP (green fluorescence; λ_ex_: 490 nm; λ_em_: 530 nm) and dimer at higher MMP (red fluorescence; λ_ex_: 520 nm; λ_em_: 590 nm). Mitochondrial depolarization was indicated by the ratio of the green/red fluorescence (λ530/λ590).

### Statistical Analysis

Data were expressed as means ± standard deprivation. For multiple groups designed experiments, comparisons were made by one-way ANOVA and followed by LSD test if variance was homogeneity, otherwise by Games-Howell test. Two-way ANOVA was applied to analyze interaction of two factors. The single effects and the main effects were analyzed by General Linear Model. All the tests were two-tailed. Statistical analysis was performed in the SPSS 16.0 statistical program (SPSS, Chicago, IL, USA). *P*<0.05 was considered to be statistically significant in the compared group.

## Supplementary Material

Supplementary Information Time of management in the experiment

## Figures and Tables

**Table 1:** Neuroprotectants screened in the study

Name	Stock Concentrations	Dissolvent	Working Concentrations	Sources
**a**lbumin(Alb)^18^	20%(solution)		20%, 5%*#,1%	Baxter
**a**trovastatin^15^	10 mM	DMSO	10,1*, 0.1 μM#	Sigma
baclofen((±)-Baclofen)^4^	10 mM	medium	1000,100*, 10 μM#	Sigma
**b**rain-derived neurotrophic factor (BDNF)^13^	1 μg/ml	medium	250,25*#,2.5 ng/ml	Sigma
bumetanide (BUM)^24^	100 mM	medium	50#,5*,0.5 μM	Sigma
citicoline sodium salt hydrate (CDPC)^22^	10 M	medium	1000,100*#,10 μM	Sigma
cyclosporin A (CsA)^20^	100 mM	DMSO	1,0.1*,0.01#μM	Sigma
deferoxaminemesylate(DFO)^52^	5 mM	medium	1000,100*#,10 μM	Sigma
disodium 4-[[(1,1,-dimethylethyl) imino]methyl] benzene-1, 3-disulfonate N-oxide (NXY-059)^12^	25 mM	medium	2.5 mM,250*#,25 μM	Selleckchem
D-α-Tocopherol succinate^10^	10 mM	ehanol	10,1*#,0.1 μM	Sigma
edaravone^9^	2.5 mM	medium	1000,100*#,10 μM	Simcere
glibenclamide(GBC)^6^	1 M	medium	1000,100*,10,1*#,0.1 μM	Sigma
gliclazide^7^	10 mM	medium	100,10*#,1 μM	Sigma
8-hydroxy-2-(n-dipropylamino) tetralin(8-OH-DPAT)^8^	10 mM	medium	1000,100*#,10 μM	Sigma
kallidinogenase (HUK human urinary)^19^	0.15 PNA/AMP	medium	0.015,0.0015*#,0.00015 PNA/AMP	Techpool
magnesium sulfate concentrate (MgSO4)^17^	0.1 M(solution)		30,3*#,0.3 M	Sigma
methylprednisolone (MP)^14^	100 mM	medium	100,10*#,1 μM	Pharmacia&Upjohn
minocycline hydrochloride (MMC)^21^	100 mM	medium	1,0.1*#,0.01 μM	Sigma
(+)-MK-801((+)-Dizocilpine hydrogen maleate (MK-801)^3^	10 mM	medium	100,10*#,1 μM	Sigma
monosialoganglioside(GM1)^23^	10 mM	methanol	10,1*#,0.1 μM	Sigma
neuroglobin (Ngb)^25^	1 μM	medium	500,50#,5 nM	Prospec
nimodipine^5^	400 μM	medium	100,10*#,1 μM	Bayer
progesterone^16^	1 mM	medium	1,0.1*#,0.01 μM	Sigma
Riluzole^53^	100 M	DMSO	1000,100*#,10 mM	Sigma
sodium pyruvate^26^	10 mM	medium	100,10*#,1 μM	Sigma
VAS2870(1,3-Benzoxazol-2-yl-3-benzyl-3H-[1,2,3]triazolo[4,5-d]pyrimidin-7-xylsulfide)^11^	10 mM	DMSO	20,2*#,0.2 μM	Sigma

*Reported working concentration; #Final working concentration for next experiment.
