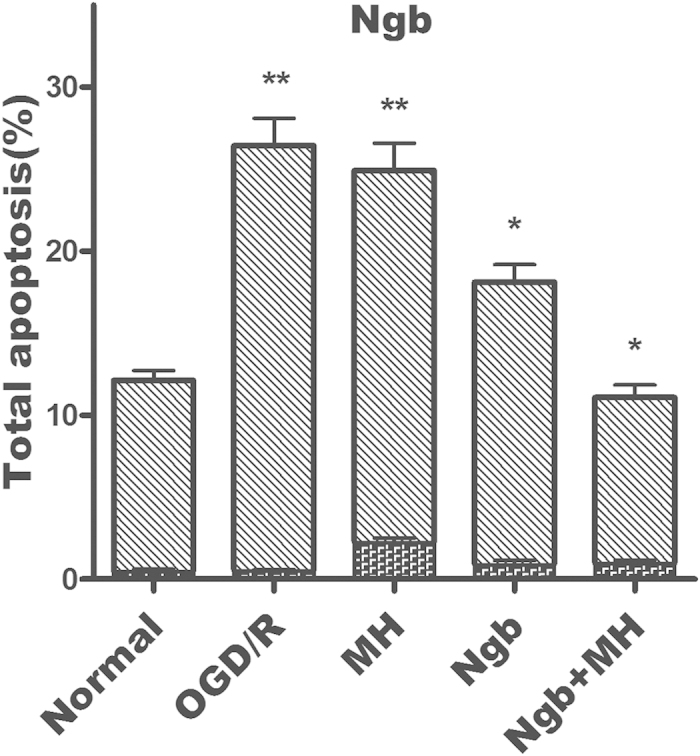# Corrigendum: Combination of mild hypothermia with neuroprotectants has greater neuroprotective effects during oxygen-glucose deprivation and reoxygenation-mediated neuronal injury

**DOI:** 10.1038/srep12195

**Published:** 2015-11-12

**Authors:** Xiao-Ya Gao, Jian-Ou Huang, Ya-Fang Hu, Yong Gu, Shu-Zhen Zhu, Kai-Bin Huang, Jin-Yu Chen, Su-Yue Pan

Scientific Reports 4: Article number: 709110.1038/srep07091; published online: 11182014; updated: 11122015

This Article contains errors in Figure 2d and Figure 2e.

In the last two histograms of Figure 2d, the y-axis should read ‘MK-801’ and ‘MK-801+MH’.

In the last two histograms of Figure 2e, the y-axis should read ‘Ngb’ and ‘Ngb+MH’.

The correct Figure 2d and Figure 2e appear below as Figure 1 and Figure 2 respectively.

## Figures and Tables

**Figure 1:**
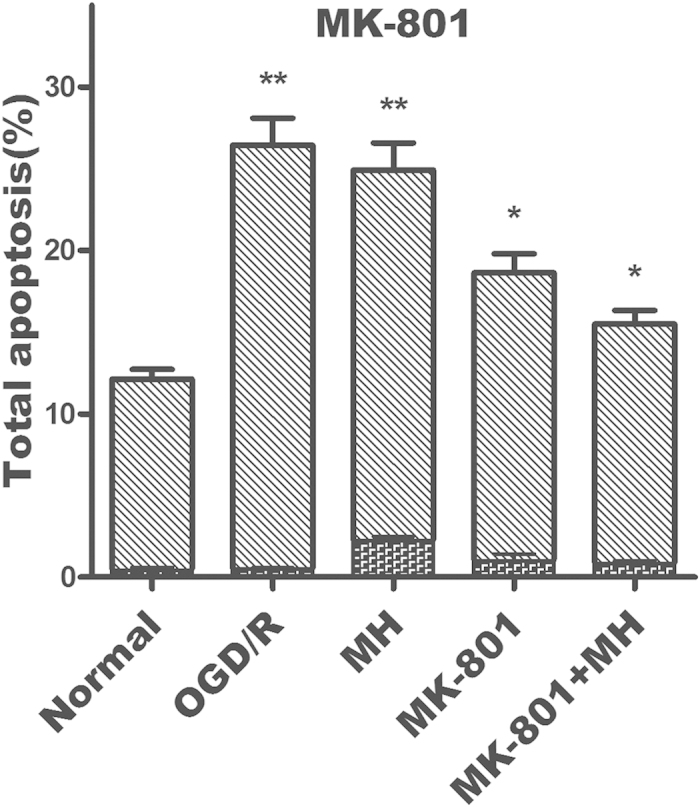


**Figure 2:**