# Supplementary material for: Combination of mild hypothermia with neuroprotectants has greater neuroprotective effects during oxygen-glucose deprivation and reoxygenation-mediated neuronal injury
Source: Sci Rep. 2014 Nov 18;4:7091. doi: 10.1038/srep07091 (PMC4665348; doi:10.1038/srep07091)
Supplement: Supplementary Information — Time of management in the experiment [file srep07091-s1.pdf]

Combination of mild hypothermiawith neuroprotectantshas greater neuroprotective effects during oxygen-glucose deprivation and reoxygenation-mediated neuronal injury

Xiao-Ya Gao[1,2], Jian-Ou Huang[1,3], Ya-Fang Hu[1], Yong Gu[1], Shu-Zhen[1], Kai-Bin Huang[1],Jin-Yu Chen[1],& Su-Yue Pan[1,*]

[1]Department of Neurology, Nanfang Hospital, Southern Medical University, Guangzhou, Guangdong, P. R. China, [2]Department of Neurology, Zhujiang Hospital, Southern Medical University, Guangzhou, Guangdong, P. R. China,&[3]Department of Neurology, the 421 Hospital, Guangzhou, Guangdong, P. R. China.

*** Corresponding Author:**

Correspondence and requests for materials should be addressed to S.Y.P (pansuyue82@126.com)

Su-Yue Pan, M.D., Ph.D., Department of Neurology, Nanfang Hospital, Southern Medical University, 1838 North Guangzhou Avenue, Guangzhou, Guangdong, 510515, P. R. China.

Tel: +86-20-61641961; Fax: +86-20-62787664

E-mail addresses: pansuyue82@126.com

Supplementary Chart 1: time of management in the experiment

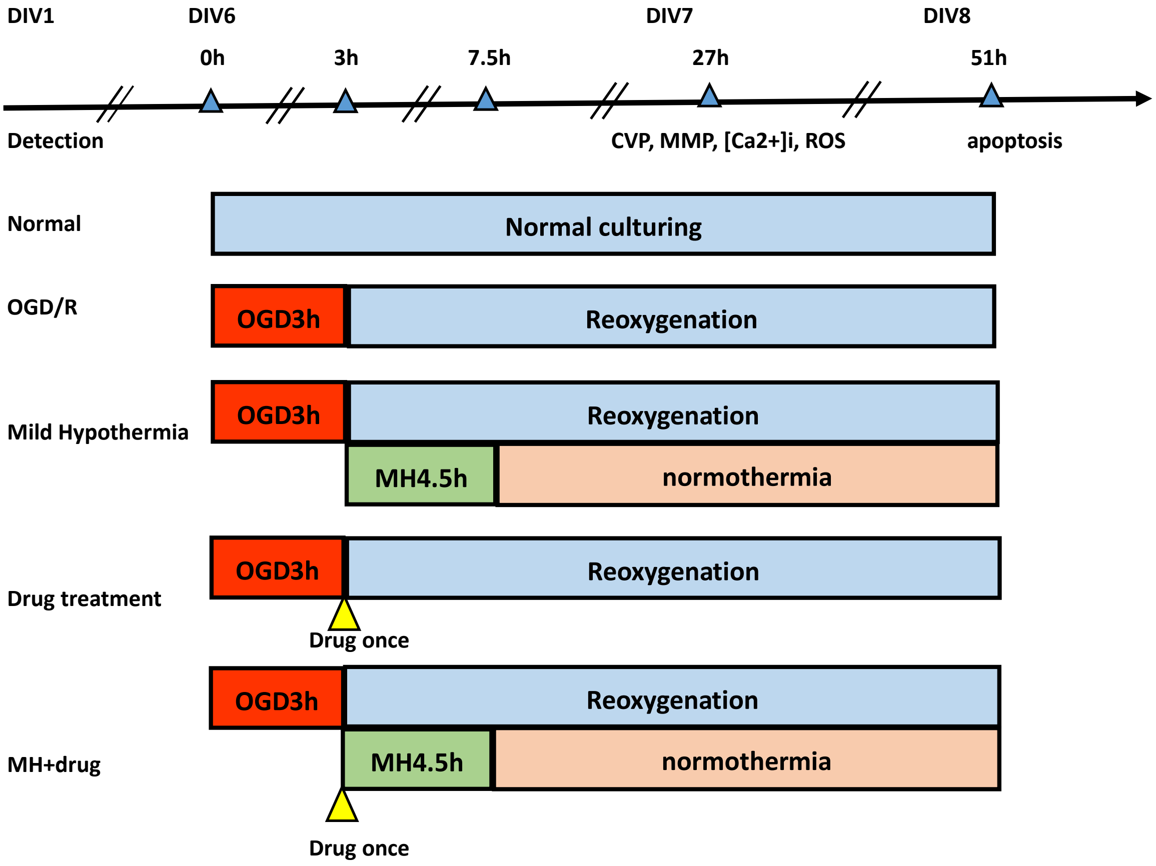

Supplementary Chart 1.

It shows the time of management in the experiment. The cultured cortical neurons (DIV 6) were subjected to OGD for 3 hrs. While glucose recovery and reoxygenation initiated, cells were immediately treated with a drug (24 hrs or 48 hrs), mild hypothermia (MH, 4.5 hrs at 34 ^{o}C, then 19.5 hrs or 43.5 hrs at 37 ^{o}C), or combination of both of them. 24 hrs after OGD (DIV 7), CVP, MMP, [Ca2+]i, or ROS were detected, and the apoptosis detected was made 48 hours after OGD (DIV8).

Abbrevation: OGD: oxygen glucose deprivation; CVP: cell viability proportion; MMP: Mitochondrial Membrane Potential; [Ca2+]i: intracellular free calcium concentration; ROS: reactive oxygen species; DIV: days in vitro.
